# Potential of Essential Oils from Anise, Dill and Fennel Seeds for the Gypsy Moth Control

**DOI:** 10.3390/plants10102194

**Published:** 2021-10-15

**Authors:** Igor Kostić, Jelica Lazarević, Darka Šešlija Jovanović, Miroslav Kostić, Tatjana Marković, Slobodan Milanović

**Affiliations:** 1 Institute for Multidisciplinary Research, University of Belgrade, Kneza Višeslava 1, 11030 Belgrade, Serbia; igork@imsi.bg.ac.rs; 2 Institute for Biological Research “Siniša Stanković”—National Institute of Republic of Serbia, University of Belgrade, Bulevar Despota Stefana 142, 11060 Belgrade, Serbia; darka.seslija@ibiss.bg.ac.rs; 3 Institute for Medicinal Plant Research “Dr Josif Pančić”, Tadeuša Košćuška 1, 11000 Belgrade, Serbia; mkostic@mocbilja.rs (M.K.); tmarkovic@mocbilja.rs (T.M.); 4 Faculty of Forestry, University of Belgrade, Kneza Višeslava 1, 11030 Belgrade, Serbia; slobodan.milanovic@sfb.bg.ac.rs or; 5 Department of Forest Protection and Wildlife Management, Faculty of Forestry and Wood Technology, Mendel University, Zemědělská 3, 613 00 Brno, Czech Republic

**Keywords:** botanical insecticide, deterrence coefficient, digestive toxicity, insect pest management, nutritional indices

## Abstract

The gypsy moth (*Lymantria dispar* L. (Lepidoptera: Erebidae)) is a serious pest of hardwood forests. In the search for an environmentally safe means of its control, we assessed the impact of different concentrations of essential oils (EOs) from the seeds of three Apiaceae plants (anise *Pimpinella anisum*, dill *Anethum graveolens*, and fennel *Foeniculum vulgare*) on behavior, mortality, molting and nutritional physiology of gypsy moth larvae (GML). EOs efficacy was compared with commercial insecticide NeemAzal^®^-T/S (neem). The main compounds in the Eos were trans-anethole in anise; carvone, limonene, and α-phellandrene in dill; and trans-anethole and fenchone in fennel seed. At 1% EOs concentration, anise and fennel were better antifeedants and all three EOs were more toxic than neem. Neem was superior in delaying 2nd to 3rd larval molting. In the 4th instar, 0.5%, anise and fennel EOs decreased relative consumption rate more than neem, whereas all three EOs were more effective in reducing growth rate, approximate digestibility and efficiency of conversion of food into body mass leading to higher metabolic costs to GML. Decrease in consumption and metabolic parameters compared to control GML confirmed that adverse effects of the EOs stem from both pre- and post-ingestive mechanisms. The results indicate the potential of three EOs to be used for gypsy moth control.

## 1. Introduction

Gypsy moth *Lymantria dispar* L. (Lepidoptera: Erebidae) is a polyphagous insect that feeds on over 500 plant species within 73 families, but the most suitable hosts are oaks (*Quercus* spp.) [1,2]. In Serbia, as well as throughout the Northern Hemisphere, gypsy moth is one of the most serious pests of hardwood forests. Its repeated outbreaks cause enormous damage to trees due to the defoliation leading to the loss of radial growth [3,4] and overall forest decline [5,6,7]. Gypsy moth outbreaks can also be very injurious in the orchards and urban green space [8,9].

The use of conventional insecticides, often in an inappropriate manner, bears the risk of evolution of insect resistance and may lead to severe environmental disturbances due to pollution and adverse effects on non-target organisms [10,11,12,13,14,15]. Having this in mind, it is not surprising that many insecticides have been removed from the market. Intensive work is being done to find methods for pest controls that are effective and, at the same time, safe for the environment [16,17,18,19,20,21]. Starting from the 1980s, broad-spectrum persistent insecticides used for gypsy moth control have been replaced with technologies based on entomopathogenic viruses, bacteria, and fungi in addition to mating disruption using sex pheromone traps [22,23]. Additionally, plant-based products have been considered as potential control agents [24,25].

Plant secondary metabolites are diverse chemical compounds synthesized through metabolic pathways derived from primary metabolism [26]. Although non-essential for plant growth and reproduction, these compounds confer protection from abiotic and biotic stressors including herbivorous insects [27]. The use of plant products for pest control has a 3000 year long history and they are considered as good candidates for environmentally safe insecticides which would negatively affect insect behavior, physiology, and life-history traits [28,29,30,31,32,33,34,35,36,37,38]. Volatile plant secondary metabolites are used by herbivorous insects to distinguish host from non-host plants [39]. The odor of volatile compounds as well as their taste provokes specific and precise behavioral responses of insects, i.e., movement away (avoidance) from the non-host or movement towards the host plant (attraction) [40]. Besides avoidance behavior induced by odor and taste of secondary plant metabolites, non-host plant compounds may also negatively affect consumption and/or impair the digestion and nutrient absorption, interfere with mitochondrial function, and have toxic, genotoxic, and prooxidant effects [41,42,43,44,45,46,47,48,49,50]. These collateral effects caused by secondary metabolites and, among them essential oils, on behavior and insect physiology are the basis for their application as botanical insecticides. High biodegradability, non or low toxicity to mammals and other non-target organisms, as well as slowed down the development of insect resistance make plant secondary metabolites far less dangerous for the environment compared to conventional insecticides [51]. 

Apiaceae species are rich in essential oils whose insecticidal and repellent activities have been confirmed in many studies on ticks, mosquitoes, cockroaches, stored products, and crop pests [38,52,53,54,55,56,57,58,59,60,61,62]. Studies on Apiaceae EOs activity against forest pests are scarce. Our previous work showed that, by spraying plants with ethanolic solutions of *Athamanta haynaldii* (Borb. Et Uecht.) the EOs provoked almost three times lower leaf damage by GML than in the control group [63]. This study was aimed to evaluate efficacy of essential oils obtained from seeds of three Apiaceae plant species (anise *Pimpinella anisum* L., dill *Anethum graveolens* L., and fennel *Foeniculum vulgare* Mill.) against GML by assessing their insecticidal and antifeeding activity as well as influence on larval molting, growth and food utilization. It was expected to find a new candidate for developing ecofriendly EO-based insecticide against GML.

## 2. Results

### 2.1. Essential Oils Chemical Composition

Essential oils (EOs) were isolated from seeds of three Apiaceae species—anise, dill, and fennel. Anise seed EO is composed of twelve compounds (one monoterpene hydrocarbon, five oxygenated monoterpenes, three phenylpropanoids, and three sesquiterpene hydrocarbons), among which the most dominant constituent was phenylpropanoid trans-anethol (Table 1, Figure S1). Sixteen compounds were present in the dill seed EO (six monoterpene hydrocarbons, nine oxygenated monoterpenes, and one sesquiterpene hydrocarbon), among which the most common ones were oxygenated monoterpene carvone and monoterpene hydrocarbons limonene and α-phellandrene. Fennel seed EO was also composed of sixteen compounds (10 monoterpene hydrocarbons, three oxygenated monoterpenes and three phenylpropanoids), but phenylpropanoid trans-anethol and oxygenated monoterpene fenchone were the major ones. High content of phenylpropanoids and oxygenated monoterpenes was detected in anise (97.66%) and fennel seed EOs (95.66%) whereas dill seed EO had lower content of oxygenated compounds (52.79%), but higher level of monoterpene hydrocarbons (Table 1).

### 2.2. Antifeeding Activity of EOs

**Figure 1:**
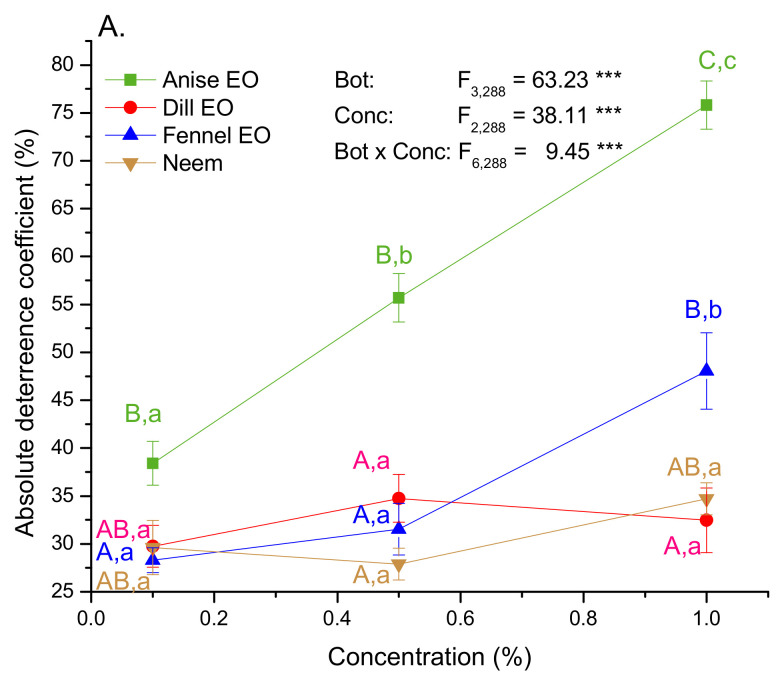

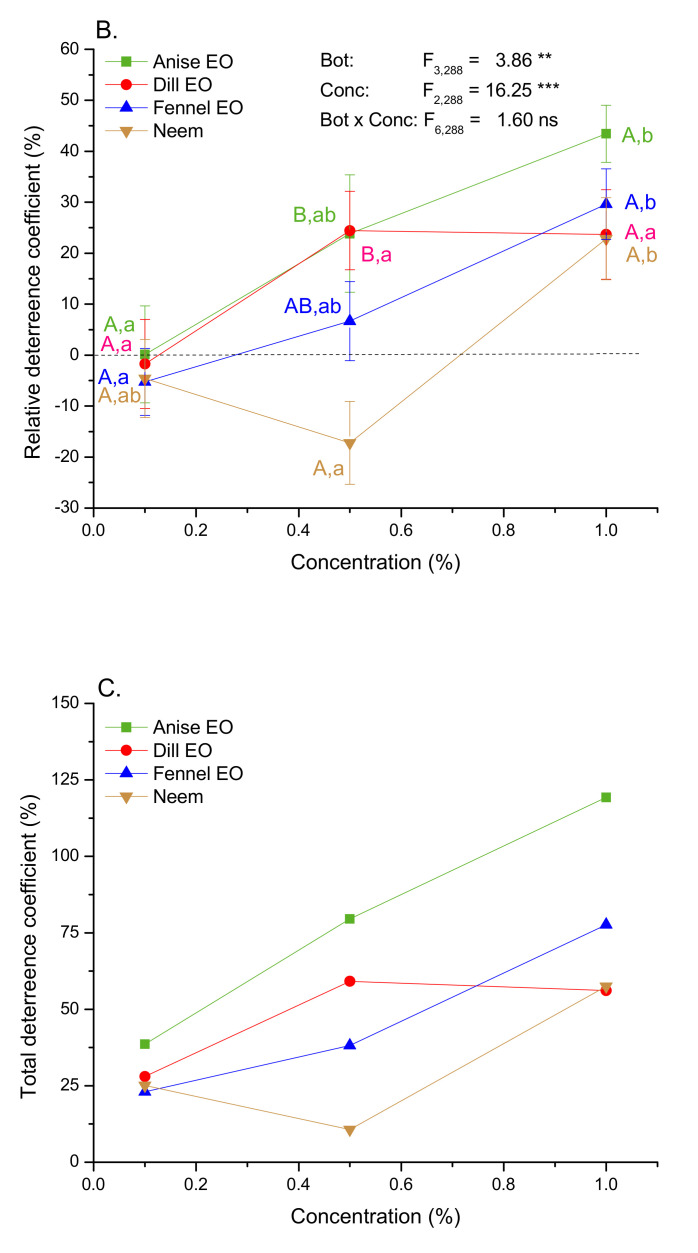
Absolute (**A**), relative (**B**) and total (**C**) deterrence coefficient (mean ± SE) in the 2nd instar GML. F-values were obtained from two-way ANOVA testing significance of the main (botanical type−Bot and concentration−Conc) and interaction (Bot × Conc) effects on analyzed traits (** *p* ˂ 0.01, *** *p* ˂ 0.001). Different colored letters mark significant differences among EOs and neem within each concentration (capital letters A, B, C), and among concentrations within each EO and neem (small letters a, b, c) (LSM contrasts, *p* ˂ 0.05).

Two-way ANOVA revealed significant effects of an applied botanical type (EOs and neem), concentration and their interaction on variation of absolute deterrence coefficient (ADC) (Figure 1A). It can be noticed that the most deterrent is anise seed EO which had the higher values of ADC than other oils and neem at all tested concentrations. The value of ADC gradually increased with concentration of anise and fennel EOs and the highest deterrence was obtained at the concentration of 1% (Tukey post hoc test for factor Conc, 0.1 vs. 0.5%: *p* = 0.003; 0.1 vs. 1% and 0.5 vs. 1%: *p* ˂ 0.001). Significant Bot × Conc interaction indicated that GML sensitivity to increasing concentration differed among EOs and neem (Figure 1A). Namely, the slope of increase in antifeeding activity with concentration was the steepest in anise EO and the most flattened in dill EO and neem. 

Antifeeding activity of the EOs was also confirmed in the choice assay. Variation in applied botanical type and in their concentration showed a significant impact on the relative deterrence coefficient (RDC) (ANOVA results in Figure 1B). Anise and dill EOs deterred larvae more effectively than neem at concentration of 0.5%. With increase in EOs and neem concentration, values of RDC changed from negative values at the lowest concentration to positive values at higher concentrations. Negative mean values of RDC recorded in experimental groups where one leaf disc was treated with 0.1% solution of dill and fennel EOs, and 0.1% and 0.5% solution of neem and 0.5% solution of neem pointed to attraction activity. 

The sum of an absolute and a relative coefficient (Tot coefficient), showed the best antifeeding activity in anise seed oil (Figure 1C). Concentration of 1% anise EO possessed good antifeeding activity (Tot = 119.26), whereas 0.5% and 0.1% solutions were average (Tot = 79.54) and weak (Tot = 38.54) antifeedants respectively. Average activity was also recorded for 1% fennel EO (Tot = 77.68), 0.5 and 1% dill EO (Tot = 59.18 and 56.12 respectively), and 1% neem (Tot = 57.52). In other experimental groups, Tot values ranged from 10.68 to 38.19 showing weak antifeeding activity.

### 2.3. Digestive Toxicity and Molting Delay Effects of EOs

**Figure 2:**
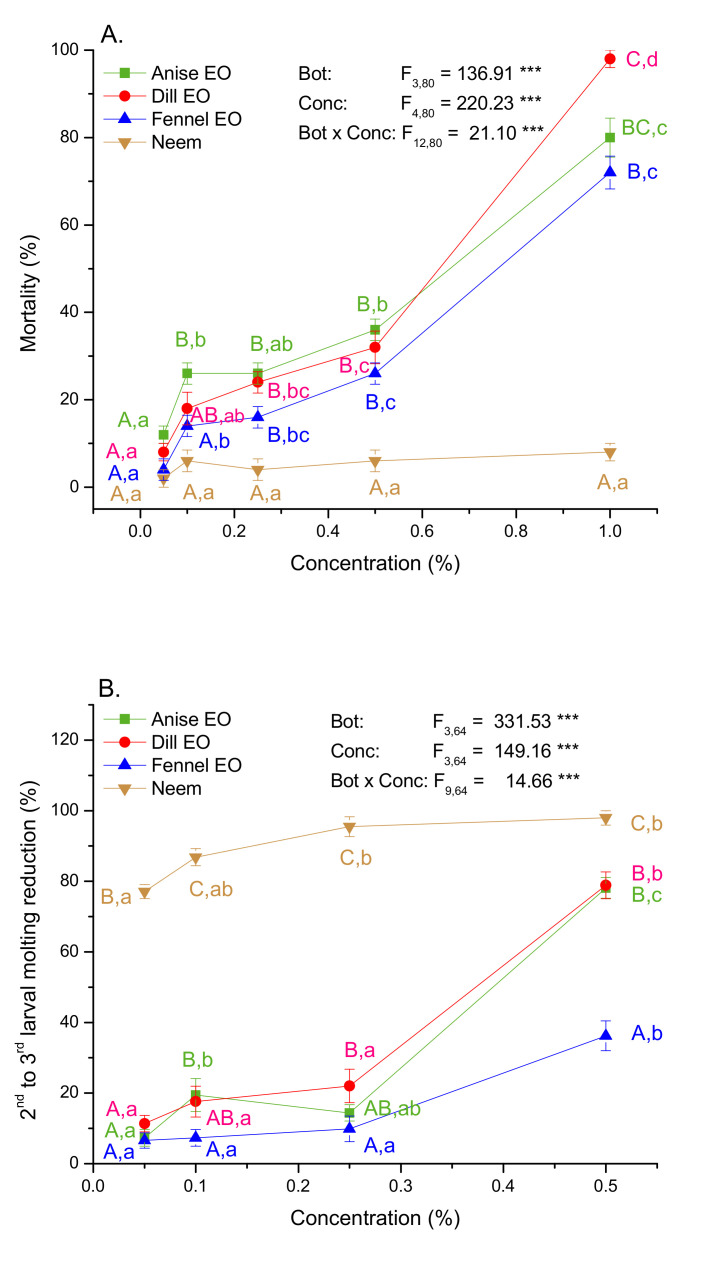
Digestive toxicity in the 2nd instar GML (**A**) and the 2nd to 3rd molting reduction (**B**) (mean ± SE) after 120 h (24 h exposure to EOs or neem supplemented diet followed by 96 h feeding on untreated diet). GML were exposed to different EOs and neem concentrations. F-values were obtained from two-way ANOVA testing significance of the main (botanical type—Bot and concentration—Conc) and interaction (Bot × Conc) effects on analyzed traits (*** *p* ˂ 0.001). Different colored letters mark significant differences among EOs and neem within each concentration (capital letters A, B, C), and among concentrations within each EO and neem (small letters a, b, c, d) (LSM contrasts, *p* ˂ 0.05). There was no mortality in GML fed on untreated diet for 120 h.

The effect of three EOs and a neem standard incorporated into the diet on larval mortality and molting was estimated after 120 h (24 h of exposure to EOs or neem followed by 96 h of feeding on control diet). As revealed by two-way ANOVA (Figure 2A) the percentage of larval mortality was significantly affected by the botanical type, as well as by the applied concentration. Comparing to neem higher toxicity was recorded in larvae fed on anise, dill, and fennel EO supplemented diets at concentrations equal or higher than 0.25%. It was also noticeable that an increase in EO concentration led to significant increase in larval mortality (Figure 2A). The significant interaction term in two-way ANOVA pointed to significantly steeper slope of mortality increase with concentration in larvae exposed to EOs than to neem. At the highest concentration (1%) dill EO was shown to be the most effective insecticide.

Larval molting into the 3rd instar was also significantly affected by the botanical type and its concentrations (ANOVA results in Figure 2B). Neem appeared to be the most effective insect growth regulator as it produced significantly the highest effect on larval molting at all tested concentrations. After 120 h, the majority of GML (98 ± 2%) was molted into the 3rd instar in the control group fed on untreated diet. Molting reducing effect of EOs and neem increased with concentration. Essential oils of anise and dill seed significantly reduced the percentage of larval molting at the concentration of 0.5% (about 78% reduction) which was similar to the effects of 0.05 and 0.1% neem that provoked 77 and 87% of reduction respectively. The highest concentration of 1% of either agent totally ceased the molting into the 3rd instar within the examined period of 120 h.

### 2.4. Impact of EOs and Neem on Mass Gain and Amounts of Consumed, Assimilated, and Metabolized Food 

**Figure 3:**
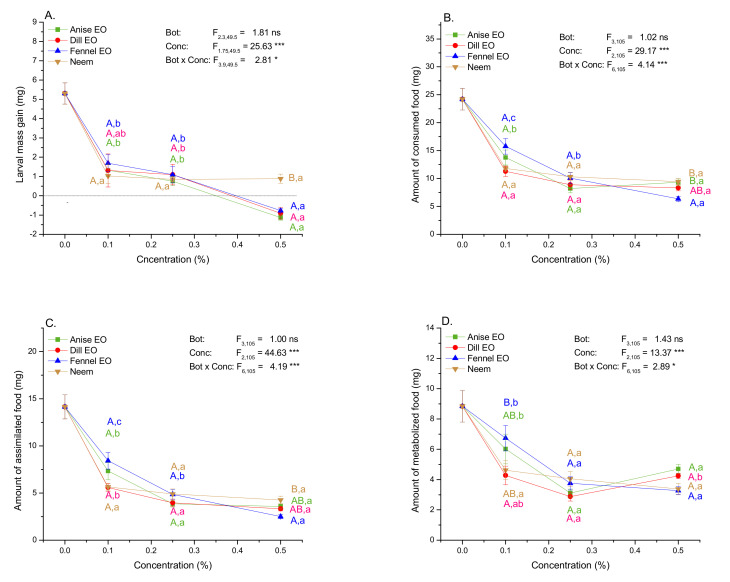
Mass gain (**A**) and amounts of consumed (**B**), assimilated (**C**) and metabolized food (**D**) (mean ± SE) in the 4th instar GML depending on the botanical type (Bot) (anise, dill and fennel EOs, commercial neem-based insecticide) and concentration (Conc). F−values indicate significance of the effects of Bot, Conc and interaction terms in two-way ANOVA (* *p* ˂ 0.05, *** *p* ˂ 0.001). Significant differences among specific experimental groups are presented by different capital colored letters A, B (EO and neem comparisons within each concentration) and small letters a, b (comparisons among concentrations within each EO and neem) (LSM contrasts, *p* ˂ 0.05).

In all treatment groups mass gain was more than 75% lower comparing to the control group (Figure 3A; one-way Welch ANOVA: F_12,43.05_ = 29.293, *p* < 0.001). Larvae exposed to EOs and neem ate 35–74% less food (Figure 3B, one-way ANOVA, F_12,113_ = 14.77, *p* < 0.001) and showed 40–82% reduction in the amount of assimilated (Figure 3C, F_12,113_ = 18.83, *p* < 0.001) and 24–67% reduction in the amount of metabolized food (Figure 3D, F_12,113_ = 6.74, *p* <0.001). Differences of treatment groups from the control group were mostly highly significant (Tables S1 and S2). 

On average, across all concentrations, EOs and neem were equally effective over these traits (Figure 3; nonsignificant Bot term in two-way ANOVAs). Negative impact became more expressed at high concentrations (significant Conc term) and it was the most apparent in larvae exposed to fennel EO. Larvae of the Neem group did not show significant change of traits with concentration increase (Figure 3; significant Bot × Conc term). In difference to neem group, larvae of the groups fed on 0.5% EOs lost their mass. In addition, larvae of the 0.5% fennel EO group larvae consumed and assimilated significantly less food. 

### 2.5. Impact of EOs and Neem on Growth and Nutritional Indices

**Figure 4:**
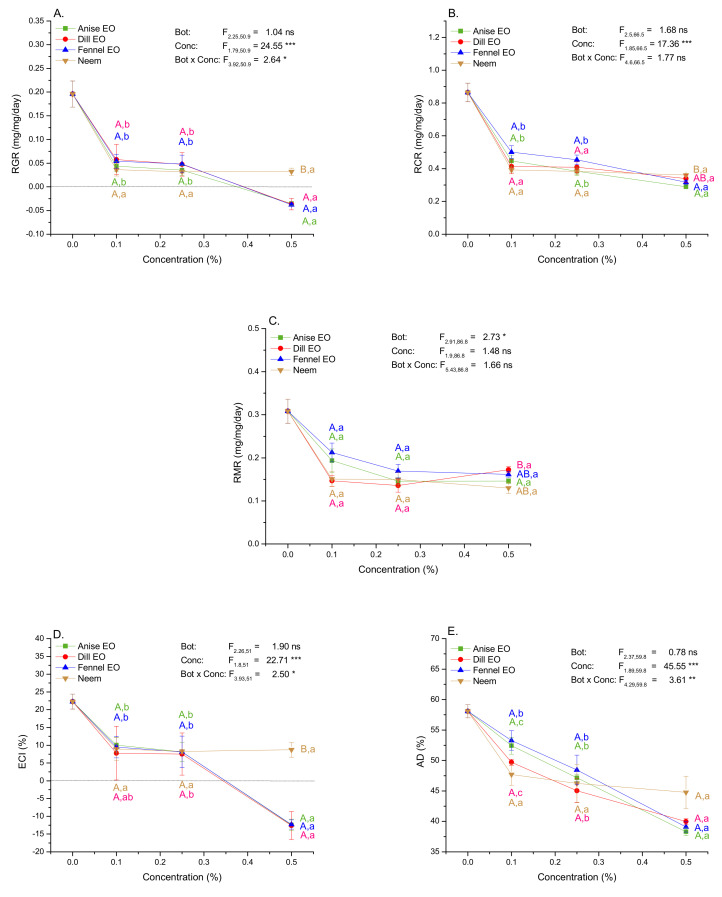

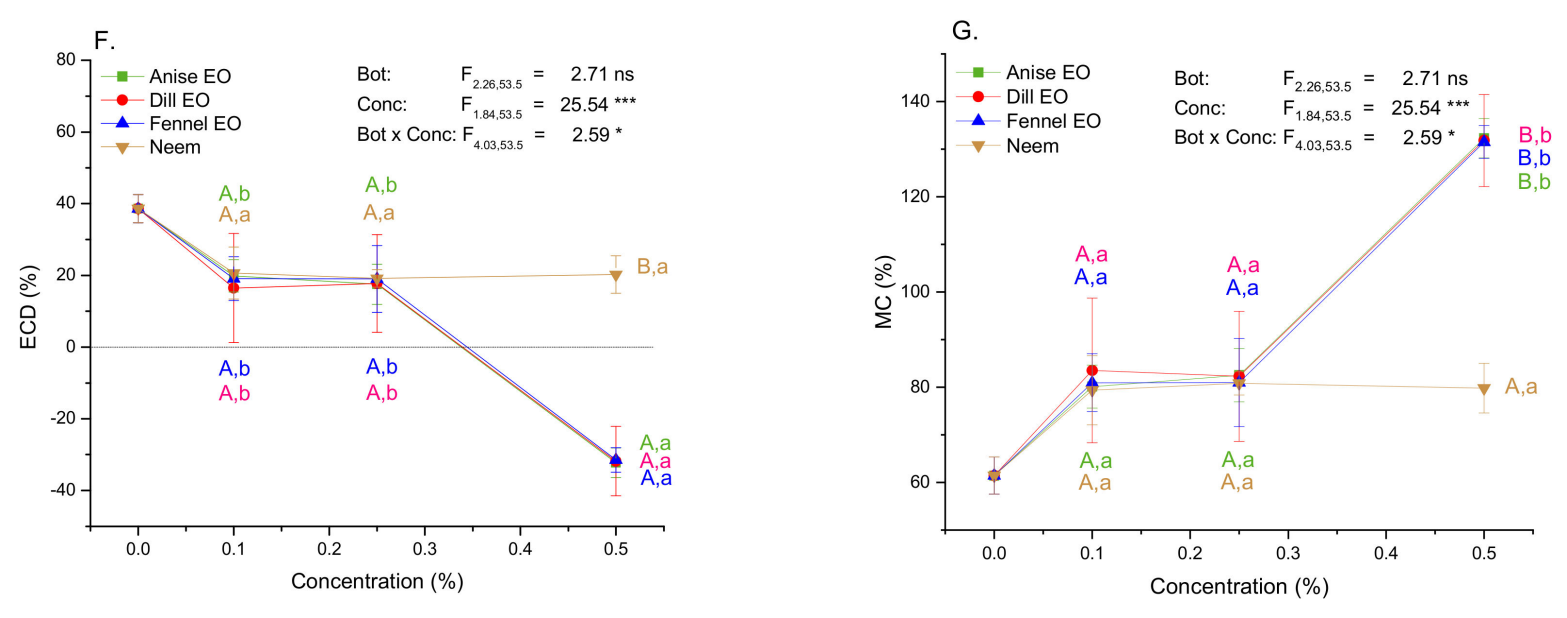
Nutritional indices (mean ± SE) in 4th instar gypsy moth larvae after 48 h of feeding on control and diet treated with EOs or neem. RGR−relative growth rate (**A**); RCR−relative consumption rate (**B**); RMR−relative metabolic rate (**C**); ECI−efficiency of conversion of ingested food (**D**); AD−approximate digestibility (**E**); ECD−efficiency of conversion of digested food (**F**); MC−metabolic cost (**G**). F−values obtained by nonparametric two-way ANOVA indicate significance of the main and interaction effects of botanical type (Bot) (anise, dill, and fennel EOs and commercial neem-based insecticide) and concentration (Conc) (* *p* ˂ 0.05, ** *p* ˂ 0.01, *** *p* ˂ 0.001). Significant differences among specific experimental groups are presented by different large colored letters A, B (EO and neem comparisons within each concentration) and small letters a, b (comparisons among concentrations within each EO and neem) (LSM contrasts, *p* ˂ 0.05).

Similar to the results of mass gain and total consumption, larval growth relative to initial mass (RGR) showed negative values and food consumption relative to initial mass (RCR) had the lowest values at 0.5% EO (Figure 4A,B). RGR (one-way Welch ANOVA, F_12,43.009_ = 31.34, *p* < 0.001) and RCR (F_12,43.068_ = 15.76, *p* < 0.001) differed significantly among experimental groups and exposure to 0.1, 0.25 and 0.5% concentration of EOs and neem led to significantly lower RGR (70–119% reduction) and RCR (42–67% reduction) values comparing to the control group (Table S2). Besides primary feeding deterrence reflected in reduced RCR, GML mostly exhibited post-ingestion toxic effects of EOs and neem as they allocate less ingested resources towards growth. In the food, EOs provoked 54–157% lower ECI values comparing to the control (F_12,42.438_ = 30.20, *p* < 0.001). Only 0.1 and 0.25% dill EO and 0.25% fennel EO acted mainly as feeding deterrents (Table S2).

Decreased ECI can be a consequence of a lower proportion of assimilated relative to consumed food (AD) and/or a lower proportion of assimilated food allocated towards growth (ECD). EOs and neem treatments significantly affected both indices (one-way Welch ANOVA, AD: F_12,43.642_ = 41.37, *p* < 0.001; ECD: F_12,43.45_ = 24.97, *p* < 0.001) leading to 8–34% and 47–184% reduction respectively. However, whereas AD was significantly reduced in the majority of EO treated groups, ECD showed significant change only at the highest EO concentration (Figure 4; Table S2). Processing food that contains 0.5% EO impose a high metabolic cost to gypsy moth larvae which exceeded the value of 100% (Figure 4G). The proportion of metabolized food relative to initial larval mass (RMR) was reduced by 31–58% (F_12,43.454_ = 4.01, *p* < 0.001) which was most probably caused by a significant decrease in the amount of assimilated food. It can be noticed from Table S2 that RMR and AD values are significantly lower comparing to the control. 

At two lowest concentrations (0.1% and 0.25%) EOs and neem did not differ in the impact on nutritional indices (Figure 4; nonsignificant Bot term in two-way ANOVA). Except for RMR, all indices were strongly dependent on concentration (Figure 4; significant Conc term in two-way ANOVA). Concentration-dependent decrease in RGR (Figure 4A), ECI (Figure 4D), AD (Figure 4E), ECD (Figure 4F), and increase in MC (Figure 4G) was steeper in EOs than neem treated groups (significant Bot × Conc term in two-way ANOVA). Comparisons of EOs impacts with the impact of neem standard at the concentration of 0.5% revealed that EOs were more effective in reducing RGR, ECI and ECD (Figure 4A,D,F). Additionally, anise and fennel EOs were more effective in reducing RCR (Figure 4B).

## 3. Discussion

The impact of essential oils on behavior, survival, and reproduction of various pest insects has been widely studied [61,64,65]. Relative effects of EOs are trait- and sex-specific, and depend on insect species, developmental stage, oil composition, mode of application, EO concentration and time of exposure [43,47,66,67,68,69]. Already recognized as potential green pesticides, EOs also proved to be promising for the management of tree pests [70,71,72,73]. In the present work, we demonstrated that EOs from three Apiaceae species (anise, dill and fennel) had significant biological activity affecting various GML traits.

### 3.1. Apiaceae EOs Are Toxic, Deter Feeding and Delay Molting in GML

Anise and dill seed EOs appeared to be the most effective agents against 2nd instar GML since anise EO had good antifeeding activity and both EOs induced high mortality and delayed larval molting. Kostić et al. [63] found strong antifeeding activity of *Athamantha haynaldii* and *Myristica fragrans* Houtt. EOs. At concentration of 0.1% these EOs showed two times higher absolute deterrence coefficient than anise EO. Additionally, *Tanacetum vulgare* L. EO delayed molting more effectively than Apiaceae EOs in our study [74]. On the other hand, digestive toxicity and antifeeding activity of Apiaceae EOs obtained in no-choice assays were higher comparing to *Ocimum basilicum* L. and *T. vulgare* EOs, and oil-in-water EO emulsions from *Thymus herba-barona* Loisel. and *Rosmarinus officinalis* L. [70,74,75,76]. The antifeeding activity of anise EO is similar to that of ethanolic leaf extracts of *Aesculus hyppocastanum* L. and *Morus alba* L. [77]. 

Our findings are in accordance with toxic, antifeeding, and molting delay effects of Apiaceae EOs described in other insect species. Anise, dill and fennel EOs had good larvicidal, repellent, and antifeeding effects on lepidopteran pests [43,47,78,79,80]. Comparisons of biological activities of anise, dill and fennel EOs revealed that relative activity depended on insect species. For example, similar to our results, dill EO is better toxicant than fennel EO in *Pseudaletia unipuncta* Haworth [81] and better toxicant than anise EO in *Musca domestica* L. larvae [82]. In contrast, fennel is more effective than dill in larvae of *Spodoptera littoralis* Boisduval [83] and more effective than anise in *Tribolium castaneum* Herbst [84]. Regarding antifeeding effects of EOs and EO compounds, many studies revealed higher deterrence in choice than no-choice assays [85,86,87]. Since we obtained higher values of the absolute than the relative deterrence coefficient, the antifeedant activity of anise, dill and fennel EOs might be more based on the post-ingestive toxicity than on their antifeeding activity. 

Comparisons of biological activity of anise, dill and fennel EOs with commercial insecticide neem revealed higher toxicity and antifeeding activity of EOs, and stronger molting delay effects of neem. It has been proved that the neem dominant component azadirachtin is very effective to inhibit the synthesis of active molting hormone 20-hydroxyecdysone, disrupts growth, may lead to incomplete ecdysis, malformations in pupae and adults and reduced fecundity [88]. EOs may also act as growth regulators and prolong the development of insect immature stages [89,90,91]. It has been suggested that components of n-butanol extracts from fruit of Apiaceae plant *Ammi visnaga* L. may inhibit ecdysone and further affect the activity of acid phosphatases and molting of *Schistocerca gregaria* Forsskål nymphs [92].

### 3.2. EO Composition Might Account for Differences in Their Biological Activity

We showed that EOs of anise and fennel were rich in phenylpropanoid trans-anethol and dill EO was rich in oxygenated monoterpene carvone and monoterpene hydrocarbons α-phellandrene and limonene. High toxicity and antifeeding activity in insect pests are induced by major compounds of anise, dill and fennel EOs: trans-anethol [93,94,95] and carvone [53,96,97,98]. It has been suggested that oxygenated compounds provide higher insecticidal activity than monoterpene hydrocarbons [99]. For example, the presence of phenylpropanoids in EOs is a significant variable determining their toxicity against *T. castaneum* [84]. In *Spodoptera frugiperda* J.E. Smith they might be involved in delayed pupation through inhibition of tyrosinase and cuticle synthesis [100,101]. However, according to our results, the most toxic to 2nd instar GML was dill EO which comparing to anise and fennel EOs contained less oxygenated compounds and more monoterpene hydrocarbons. Studies on the structure-function relationship of EO constituents point out that molecular shape, degree of saturation, volatility and type of functional groups contribute to the efficacy of natural insecticides [102]. Besides, it has been well documented that EO compounds act in synergy and affect multiple targets in pest insects [93,94,103,104,105].

Physiological mechanisms of insecticidal activity of EOs, their compounds and/or a mixture of compounds is to act as neurotoxins and lead to the paralysis and death of insect pests through the inhibition of acetylcholine esterase (AChE), as well as through blockage of octopamine receptors and/or interference with GABA-gated sodium channels [64]. For example, anise and fennel EOs as well as their constituents, anethol, phellandrene, limonene, fenchone, carvon, and estragol inhibit AChE, carvon intensify GABA-induced Cl- current whereas limonene acts through the octopaminergic system [84,106,107,108,109]. Modulation of the GABA-ergic system by plant secondary metabolites is related to their antifeeding activity in pest insects [110,111,112]. Besides modulation of odor and gustatory receptors, the antifeeding activity of EOs can be also an indirect consequence of induced toxicity due to disrupted structure of midgut peritrophic membrane and epithelium, oxidative damages to macromolecules, and inhibition of digestive and detoxification enzymes [44,47,113,114].

### 3.3. Apiaceae EOs Reduce GML Growth through Pre- and Post-Ingestive Mechanisms

Obviously, exposure to EOs and EOs compounds induce numerous changes in physiological processes that may further affect pest insect behavior and life-history traits. In the present paper, we assessed how three Apiaceae EOs affected the growth and nutritional indices of GML. We showed mainly significant adverse effects of EOs and neem on GML growth (RGR), consumption (RCR), assimilation (AD) and metabolism (RMR, ECI, ECD, MC). In difference to neem experimental group, larvae fed on 0.5% EO supplemented diet lost their mass and had negative values of growth and gross/net growth efficiencies (RGR, ECI, ECD). Therefore, GML mass change during 2 days of feeding was provoked not only by among-treatment variation in the amount of consumed and assimilated food (pre-ingestive and pre-digestive mechanisms, respectively) but also was a consequence of the EO influence on post-ingestive and post-digestive mechanisms. Our results are in accordance with findings of other studies which showed negative values of RGR and ECI in *T. castaneum* adults exposed to anise EO [45] and 4th instar *P. unipuncta* larvae exposed to trans-anethol [55]. 

Reduced consumption obtained in the present paper in 2nd and 4th instars fed on anise, dill and fennel EO treated diets as well as in studies on the effects of other EOs on GML [63,70,74,75] pointed to the sensitivity of this species to the presence of antifeedants in EOs. Many papers also confirmed the antifeedant activity of terpenes and terpenoids in GML [49,115,116]. Comparing to tansy EO [74], Apiaceae EOs appeared to be more effective in reducing GML growth and consumption. Tansy EO did not induce mass loss and negative values of ECI and ECD, consumption was decreased by 38% compared to 63–67% reduction in response to Apiaceae EOs. In addition, AD was not affected by tansy EO whereas 31–34% and 23% reduction was recorded on Apiaceae EOs and neem respectively. EOs [44,114] and azadirachtin [117,118,119,120] disrupted gut structures and thus inhibited digestive enzyme activities and impaired nutrient absorption which might account for the observed decrease in ECI and AD. 

Our results showed that RMR decreased along with a decrease in RCR. Such response depended on concentration so that RMR and RCR were not significantly changed at the lowest concentration of anise and fennel EOs. Likewise, extracts of *Inula racemosa* Hook which contained sesquiterpene lactones did not affect RCR and RMR of *Spodoptera litura* Fabricius larvae at the lowest examined concentration, whereas both indices were reduced at the highest concentration [121]. Sousa et al. [65] recorded a significant reduction in RMR in *P.* *unipuncta* exposed to trans-anethol and EO from *Petroselinum crispum* (Mill.) Nyman ex A.W. Hill, two botanicals which induced larval mass loss. Despite the decrease in the amount of metabolized food in GML fed on EO- and neem-supplemented diets, its proportion relative to ingested and assimilated food increased leading to lower ECI and ECD values.

Reduction in food utilization efficiency by botanical treatments points to their chronic toxicity which forces larvae to reallocate energy resources from growth to defense. Depletion of metabolites and adaptive increase in antioxidative and detoxification enzymes have been described in pest insects exposed to EOs [114,122,123,124,125,126] and azadirachtin [127,128]. Similar results were obtained in studies dealing with the impact of Apiaceae EOs on insect pests [45,113,129,130,131]. In lepidopteran pests, *S. frugiperda* and *Anticarsia gemmatalis* Hübner limonene, carvon, estragol and anethol (compounds of anise, dill and fennel seed EOs) are detoxified by microsomal cytochrome P-450 monooxygenases [132]. This enzyme is also elevated in GML fed on terpene-rich plants [133].

## 4. Materials and Methods

### 4.1. Plant Material and EOs Isolation 

Tested EOs have been extracted from seeds of anise (*Pimpinella anisum* L.), dill (*Anethum graveolens* L.), and fennel (*Foeniculum vulgare* Mill.) cultivated on the experimental fields of the Institute for Medicinal Plant Research “Dr. Josif Pančić” in Pančevo, Serbia (44°52′14″ N; 20°38′42.7164″ E, altitude 81 m). EOs were obtained by hydrodistillation of their seeds using a Clevenger-type apparatus [134].

### 4.2. Chemical Characterization of EOs

The essential oils samples were diluted in ethanol (10 μL mL^−1^) and 1 μL of each solution was injected in a split-mode (1:30). Gas chromatography was performed using the GC Agilent Technologies 7890A apparatus equipped with a split-splitless injector attached to an HP-5 column (30 m × 0.32 mm, film thickness 0.25 μm) and fitted to a flameionization detector (FID). The operating conditions were: the carrier gas was H2 (1 mL/min/210 °C); the temperatures were set as follows: injector at 250 °C and detector at 280 °C, while the column temperature was linearly programmed from 40 to 260 °C at 4 °C/min. The percentage composition was computed from the peak areas, without correction factors. 

The Gas Chromatography—Mass Spectrometry was performed using the HPG 1800 C Series II GCD analytical system equipped with an HP-5MS column (30 m × 0.25 mm, film thickness 0.25 m). The carrier gas was He (1 mL/min). Other chromatographic conditions were the same as those for Gas Chromatography with Flame-Ionization Detection. The transfer line was heated at 260 °C. The mass spectra were recorded in the EI mode (70 eV) in the range of *m*/*z* 40–450. The identification of individual constituents was accomplished by comparing their spectra to those available from MS libraries (NIST/Wiley) and by comparing their experimentally determined retention indices (calibrated AMDIS) to the data from the literature [135].

### 4.3. GML Rearing

Gypsy moth egg masses were collected from natural populations in the Lipovica Forest, near Belgrade, Serbia (44°38′34″ N; 20°26′13″ E, altitude 270 m) during the autumn. Egg masses were maintained at 4 °C until the next spring. Eggs were mechanically cleaned of hairs, disinfected by soaking into 0.1% sodium hypochlorite solution for 5 min, washed with distilled water for 10 min and air-dried [136]. Eggs from the middle parts of 25 egg masses (100 eggs per egg mass, 2500 eggs in total) were mixed and put into flasks for hatching in a SANYO microclimate chamber at 25 ± 1 °C, 65 ± 5% relative humidity and neon diffuse light of 30159.29 candelas with a 15:9 L:D photoperiod. Newly hatched larvae were transferred to Petri dishes (90 × 14 mm) at a density of ten 1st instar larvae per dish and fed with an artificial gypsy moth diet (MP Biomedicals, Inc., Irvine, CA, USA, cat. no. 296029304). 

### 4.4. Antifeeding Activity 

The antifeeding activity was assessed in the 2nd instar larvae by no-choice and choice tests. After the molting into the 2nd instar, larvae were starved for 24 h. An agar-water (2%) layer of 2 mm thickness was poured into Petri dishes (90 × 14 mm). After agar turned solid we covered it with wet filter paper and placed one oak (*Quercus robur* L.) leaf disc (30 mm diameter) in the center of the Petri dish in the no-choice test or two discs on opposite sides of the Petri dish in the choice test. Leaf disc treatments were performed by the leaf dipping method [137]. Namely, discs were immersed either in 50% ethanolic solution of EOs or NeemAzal^®^-T/S (Trifolio-M GmbH) at three different concentrations (0.1, 0.5, and 1.0%) or in a solvent for 3 s. In the no-choice test leaf discs were treated with the EOs, neem or solvent, whereas in the choice test, one leaf disc was treated with the EOs or neem and the other with the solvent. After 30 minutes’ evaporation of the solvent, leaf discs were fixed to the agar layer with pins. Then one larva was introduced into the center of each Petri dish. After 48 h, the remains of the consumed discs were scanned at 200 dpi in jpg format. Quantification of the consumed surface area for each leaf disc was done by subtracting remained leaf disc area from the disc area at the start of the experiment using ImageTool software 3.0 [138]. In each experimental group antifeeding activity of EOs and neem was analyzed in 25 larvae (replicates). 

Based on the consumed leaf disc areas in the no-choice and choice tests, absolute (ADC), relative (RDC), and total (Tot) deterrence coefficients were calculated according to the formulas of [85,139]:

ADC = (CC − TT)/(CC + TT) × 100(1)

RDC = (C − T)/(C + T) × 100(2)

Tot = A + R(3)

where CC is the mean consumed surface area for GML from the control group and TT is the consumed surface area of the leaf discs treated with EOs or neem in the no-choice test; C is the consumed surface area of the control leaf disc and T is the consumed surface area of the treated leaf disc in the choice test.

The total deterrence coefficient can range from −200 to +200. According to Tot values EOs can be ranged as very good (Tot values from 151 to 200), good (101–150), average (51–100), and weak deterrents (˂50). Negative Tot values suggest attractant properties of EOs. 

### 4.5. Digestive Toxicity and Molting 

In the digestive toxicity test, EOs and neem were incorporated into the artificial diet at different concentrations (0—control group, 0.05, 0.1, 0.25, 0.5, and 1%). After starvation for 24 h, ten 2nd instar larvae were put into the Petri dishes and fed ad libitum either on control diet or on EOs and neem treated diets for 24 h. After that, they were transferred into clean Petri dishes and fed on control diets for another 96 h (120 h from the beginning of exposure). During the experiment, two fresh cubes of artificial diet per Petri dish were provided daily. Within each EO or neem and their concentration five replicates were analyzed (5 × 10 larvae per experimental group). Larval mortality and molting were monitored daily and 120 h after the beginning of experiment, the percentage of mortality and percentage of molting into the 3rd larval instars were determined. The percentage of molting reduction relative to the control was calculated as (C − T)/C × 100 where C was the mean percentage of molted larvae in the control group and T was the percentage of molted larvae fed on EO or neem treated diet.

### 4.6. Growth and Nutritional Indices 

After molting into the 4th larval instar, larvae were separated and exposed to 24 h starvation after which their mass was measured individually. Larvae were daily supplied with cubes of the artificial diet with incorporated EOs or neem. As preliminary study showed that larvae fed on EO supplemented diets decreased their mass at the concentration of 0.5%, the study of EO and neem influence on nutritional physiology of GML encompassed concentrations of 0 (control), 0.1, 0.25, and 0.5%. Cubes of artificial gypsy moth diet were weighed before and after the feeding trial, as well as the excrement was weighed at the end of the experiment. Larval mass was measured again 48 h after the experiment was set. All indices were estimated on a dry mass basis. Larvae, uneaten cubes and excrements were dried at 65 °C for 72 h. After this time, the mass of each larva, uneaten cubes, and excrements were weighed. A regression of dry on fresh mass in a random sample of 30 larvae and cubes of artificial diet per experimental group was used for estimating the dry mass of larvae and cubes of artificial diet at the beginning of the experiment. Based on these data the following indices were calculated according to the standard formulas [140,141,142] (Table 2).

### 4.7. Statistical Analysis

Parametric one-way and two-way ANOVAs were performed by software package Statistica 7.0 (StatSoft, Inc., Tulsa, OK, USA) on untransformed values of the relative deterrence index and (X + 0.5)^0.5^ transformed values of the absolute antifeeding index, larval mortality, larval molting reduction, and log-transformed values of the amount of consumed food, amount of assimilated food, and amount of metabolized food. Dunnett test following one-way ANOVA was used to estimate the significance of differences of treatment groups from the control group. Two-way ANOVA was carried out to evaluate the main and interaction effects of the botanical type (EOs or neem) and the botanical concentration as fixed factors on examined traits. A least square means (LSM) test with the Bonferroni correction was used for a posteriori comparisons (contrasts) of different concentration effects within each EO and neem as well as comparisons of EOs and neem effects within each concentration. 

For mass gain, and growth and nutritional indices assumption of homogeneity of variances was strongly violated (Levene’s test, *p* ˂ 0.0001). Therefore, significant differences of treatment groups from the control group were revealed by Welch one-way ANOVAs for each pair of comparison followed by the Bonferroni correction [143]. Significance of main and interaction effects of botanical type and concentration on growth and nutrition indices were estimated by nonparametric two-way ANOVA [144]. LS means tests with Bonferroni correction was used for a posteriori comparisons.

## 5. Conclusions

In whole, Apiaceae EOs can be considered as promising strategy for gypsy moth control based on strong negative effects on survival and consumption in 2nd instar, and impairment of nutritional physiology in 4th instar larvae. Whereas anise EO was the best antifeedant, dill EO exhibited the highest mortality. At a concentration of 0.5%, the three Apiaceae EOs were more effective than commercial insecticide neem in reducing RGR, ECI, AD and ECD of 4th instar GML. Anise, dill and fennel are spice plants used in human nutrition, and have low toxicity to mammals and other non-target organisms [43,103,145,146,147,148]. However, the commercialization of anise and dill EO-based insecticides necessitates further investigations to find formulations of improved EO solubility and persistence whose efficacy would be finally tested in the field [19].

## Figures and Tables

**Table 1:** Chemical composition of essential oils obtained from seeds of anise (*Pimpinella anisum*), dill (*Anethum graveolens*) and fennel (*Foeniculum vulgare*). Compounds are grouped according to their chemical class and total portion of each class is presented in bold. Contents of major compounds are also given in bold. RI_lit_—Kovats retention indices; RI_exp_—values for retention indices on the HP-5 column; tr—contents < 0.05%.

RI_lit_	RI_exp_	Compound	Contribution to Essential Oil (% m/m)		
			Anise	Dill	Fennel
		***Monoterpene hydrocarbons***	***0.61***	***45.50***	***4.12***
921	919	Tricyclene	-	0.13	-
924	924	α-Thujene	0.61	0.44	-
932	924	α-Pinene	-	-	1.53
946	938	Camphene	-	-	0.16
969	965	Sabinene	-	-	tr
974	967	β-Pinene	-	-	0.13
988	986	β-Myrcene	-	0.51	tr
1002	996	α-Phellandrene	-	**13.12**	0.38
1008	1003	δ-3-Carene	-	-	tr
1020	1018	*p*-Cymene	-	2.26	0.28
1024	1021	Limonene	-	**29.04**	1.32
1054	1052	γ-Terpinene	-	-	0.32
		***Oxygenated monoterpenes***	***4.75***	***52.79***	***26.38***
1026	1024	1,8-cineole	2.35	-	0.17
1083	1080	Fenchone	-	-	**25.56**
1095	1097	Linalool	0.43	-	-
1141	1135	Camphor	-	-	0.65
1148	1146	Menthone	-	0.42	-
1158	1156	*iso*-Menthone	-	0.17	-
1161	1166	*neo*-Menthol	-	0.28	-
1174	1172	Terpinen-4-ol	0.05	-	-
1184	1176	Dill ether	-	6.52	-
1186	1185	α-Terpineol	0.22	-	-
1191	1189	*cis*-Dihydrocarvone	-	1.52	-
1200	1196	*trans*-Dihydrocarvone	-	0.81	-
1212	1210	*iso*-Dihydrocarveol	-	0.14	-
1226	1223	*neoiso*-Dihydrocarveol	-	0.46	-
1239	1238	Carvone	-	**42.47**	-
1380	1388	Anisyl methyl ketone	1.70	-	-
		***Phenylpropanoids***	***92.91***	***0.00***	***69.28***
1195	1193	Methyl chavicol (estragole)	5.32	-	3.44
1249	1248	*cis*-Anethol	0.11	-	0.79
1282	1280	*trans*-Anethol	**87.48**	-	**65.05**
		***Sesquiterpene hydrocarbons***	***1.41***	***0.24***	***0***
1374	1366	α-Copaene	tr	-	-
1400	1397	β-Longipinene	0.10	-	-
1471	1470	Dauca-5,8-diene	-	0.24	-
1500	1480	γ-Himachalene	1.31	-	-
		Total identified	99.68	98.53	99.78

**Table 2:** Formulae for calculation of growth and nutritional indices.

Indices	Formula
Relative growth rate	RGR = (m_2_ − m_0_)/(2 × m_0_)
Relative consumption rate	RCR = m_c_/(2 × m_0_)
Relative metabolic rate	RMR = [(m_c_ − m_e_) − (m_2_ − m_0_)]/(2 × m_0_)
The efficiency of conversion of ingested food (gross growth efficiency)	ECI = (m_2_ − m_0_)/m_c_ × 100
Approximate digestibility (assimilation efficiency)	AD= (m_c_ − m_e_)/m_c_ × 100
The efficiency of conversion of digested food (net growth efficiency)	ECD = (m_2_ − m_0_)/(m_c_ − m_e_) × 100
Metabolic cost	MC = 100 − ECD

m_0_—initial larval mass at the beginning of the experiment; m_2_—larval mass at the end of the experiment (2 days of feeding); (m_2_ − m_0_)—mass gain; 2—duration of the experiment expressed in days; m_c_—the amount of consumed food (difference between final and initial dry food mass); m_e_– a dry mass of excrements; (m_c_ − m_e_)—the amount of assimilated food; [(m_c_ − m_e_) − (m_2_ − m_0_)]—the amount of metabolized food.

## Data Availability

Publicly available datasets were analyzed in this study. This data can be found here: http://radar.ibiss.bg.ac.rs/handle/123456789/4465, accessed on 12 October 2021.

## Supplementary Materials

The following are available online at https://www.mdpi.com/article/10.3390/plants10102194/s1, Table S1: Summary of *p*-values from Dunnett test indicating significance (values in bold) of differences in amounts of consumed, assimilated and metabolized food between treatment groups and control group. Table S2: Summary of *p*-values from the Welch one-way ANOVA indicating significance (values in bold) of differences in growth and nutritional indices between treatment groups and control group. MG—mass gain; RGR—relative growth rate; RCR—relative consumption rate; RMR—relative metabolic rate; ECI—efficiency of conversion of ingested food; AD—approximate digestibility; ECD—efficiency of conversion of digested food; MC—metabolic cost. Figure S1: Chromatograms obtained for the EOs extracted from the seeds of Apiaceae plants (anise—*Pimpinella anisum*, dill—*Anethum graveolens*, fennel—*Foeniculum vulgare*).
