# Supplementary material for: Potential of Essential Oils from Anise, Dill and Fennel Seeds for the Gypsy Moth Control
Source: Plants (Basel). 2021 Oct 15;10(10):2194. doi: 10.3390/plants10102194 (PMC8538750; doi:10.3390/plants10102194)
Supplement: Supplementary file 1 [file plants-10-02194-s001.zip › Table S1.pdf]

Table S1. Summary of *p* values from Dunnett test indicating significance (values in bold) of differences in amounts of consumed, assimilated and metabolized food between treatment groups and control group.

	Amount of food		
	**Consumed**	**Assimilated**	**Metabolized**
Anise 0.1%	**<0.001**	**<0.001**	0.134
0.25%	**<0.001**	**<0.001**	**<0.001**
0.5%	**<0.001**	**<0.001**	**0.010**
Dill 0.1%	**<0.001**	**<0.001**	**<0.001**
0.25%	**<0.001**	**<0.001**	**<0.001**
0.5%	**<0.001**	**<0.001**	**0.003**
Fennel 0.1%	**0.006**	**0.004**	0.650
0.25%	**<0.001**	**<0.001**	**<0.001**
0.5%	**<0.001**	**<0.001**	**<0.001**
Neem 0.1%	**<0.001**	**<0.001**	**0.002**
0.25%	**<0.001**	**<0.001**	**<0.001**
0.5%	**<0.001**	**<0.001**	**<0.001**
