# Supplementary material for: Potential of Essential Oils from Anise, Dill and Fennel Seeds for the Gypsy Moth Control
Source: Plants (Basel). 2021 Oct 15;10(10):2194. doi: 10.3390/plants10102194 (PMC8538750; doi:10.3390/plants10102194)
Supplement: Supplementary file 1 [file plants-10-02194-s001.zip › Table S2.pdf]

Table S2. Summary of *p* values from the Welch 1-way ANOVA indicating significance (values in bold) of differences in growth and nutritional indices between treatment groups and control group. MG- mass gain; RGR- relative growth rate; RCR- relative consumption rate; RMR- relative metabolic rate; ECI- efficiency of conversion of ingested food; AD- approximate digestibility; ECD- efficiency of conversion of digested food; MC- metabolic cost.

	**MG**	**RGR**	**RCR**	**RMR**	**ECI**	**AD**	**ECD/MC**
Anise 0.1%	**<0.001**	**0.005**	**<0.001**	0.112	**0.011**	0.065	0.074
0.25%	**<0.001**	**0.003**	**<0.001**	**0.004**	**0.008**	**<0.001**	0.086
0.5%	**<0.001**	**<0.001**	**<0.001**	**0.004**	**<0.001**	**<0.001**	**<0.001**
Dill 0.1%	**0.019**	0.057	**<0.001**	**0.003**	1.000	**<0.001**	1.000
0.25%	**<0.001**	**0.011**	**<0.001**	**0.002**	0.463	**<0.001**	1.000
0.5%	**<0.001**	**<0.001**	**<0.001**	**0.014**	**<0.001**	**<0.001**	**<0.001**
Fennel 0.1%	**0.001**	**0.007**	**0.001**	0.196	**0.039**	0.292	0.199
0.25%	**<0.001**	**0.006**	**<0.001**	**0.002**	0.156	**0.042**	0.906
0.5%	**<0.001**	**<0.001**	**<0.001**	**0.009**	**<0.001**	**<0.001**	**<0.001**
Neem 0.1%	**<0.001**	**0.003**	**<0.001**	**0.004**	**0.047**	**0.002**	0.570
0.25%	**<0.001**	**0.003**	**<0.001**	**0.004**	**0.001**	0.050	**0.011**
0.5%	**<0.001**	**0.003**	**<0.001**	**0.001**	**0.004**	**0.009**	0.159
