# Supplementary figures and images for: Potential of Essential Oils from Anise, Dill and Fennel Seeds for the Gypsy Moth Control
Source: Plants (Basel). 2021 Oct 15;10(10):2194. doi: 10.3390/plants10102194 (PMC8538750; doi:10.3390/plants10102194)

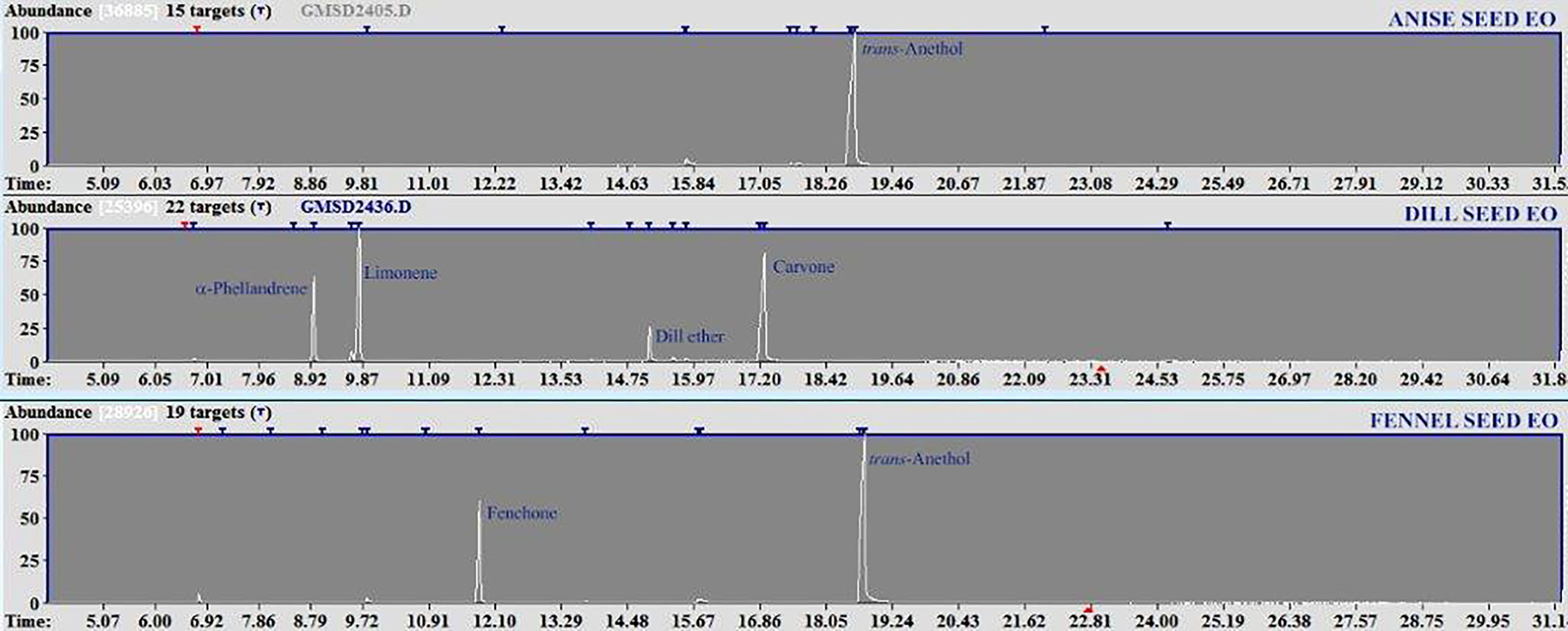

Supplement: Supplementary file 1 [file plants-10-02194-s001.zip › Figure S1.jpg]
